# Supplementary material for: Parental investment and immune dynamics in sex-role reversed pipefishes
Source: PLoS One. 2020 Sep 25;15(9):e0228974. doi: 10.1371/journal.pone.0228974 (PMC7518610; doi:10.1371/journal.pone.0228974)
Supplement: S1 Protocol — Details on Preamplification of target cDNA, sample mix and assay mix and GE-chip loading technique. (DOCX) [file pone.0228974.s003.docx]

Supplementary Protocol 1:

Gene Expression of 48 genes using a Fluidigm-BioMarkTM system based on 96.96 dynamic arrays (GE-Chip).

Pre-amplification of target cDNA:

2.5 µl TaqMan PreAmp Master Mix (Applied Biosystems),

0.5 µl of 500nM combined primer pairs (diluted with TE Buffer)

0.75 µl HPLC H_2_O

1.4 µl of cDNA.

Mixture was pre-amplified (1x 10 min; 95 °C; 16x (15 s; 95 °C, 4min; 60 °C)) and diluted 1:10 with low EDTA-TE Buffer.

For the chip run:

Sample mix

3.5 µl 2x SSo FastEvaGreen Supermix with low Rox (BioRad)

0.37 µl 20x DNA binding Dye sample loading reagent (Fluidigm)

3.3 µl of pre-amplified 1:10 diluted cDNA

Assay mix

3.5 µl 2x Assay loading reagent (Fluidigm)

3.15 µ 1x low EDTA-TE Buffer on

0.7 µl of 50 µM Primer mix have been prepared.

5 µl of each mix were loaded on a GE-chip, and measured with the GE-fast 96.96 PCR protocol in the BioMarkTM system according to Fluidigm instructions.

In each Chip run we included two technical replicates, a negative control (HPLC H_2_O) and a –RT control to test for residual gDNA
